# Seismic Performance of Recycled Aggregate Concrete-Filled Steel Tube Column–Composite Beam Frames with Column-End Stirrup Confinement

**DOI:** 10.3390/ma18112458

**Published:** 2025-05-23

**Authors:** Zhi Yang, Xingnian Chen, Hongchang Xu, Baoye Hui, Jia Huang, Liping Wang, Said Ikram Sadat, Faxing Ding

**Affiliations:** 1 School of Civil Engineering, Central South University, Changsha 410075, China; csjthr2024@163.com (Z.Y.); huangjiacsu@163.com (J.H.); saidikramsadat@hotmail.com (S.I.S.); dinfaxin@csu.edu.cn (F.D.); 2 China Construction Fourth Engineering Division Co., Ltd., Guangzhou 510630, China; dwz2520226@163.com; 3 China Construction Third Engineering Bureau Co., Ltd., Wuhan 430064, China; xuhongchang521@163.com (H.X.); zjsj_huibaoye@163.com (B.H.); 4 National Engineering Research Center of High Speed Railway Construction Technology, Changsha 410075, China

**Keywords:** recycled aggregate concrete, seismic performance, concrete-filled steel tube (CFST) structures, column-end stirrup confinement

## Abstract

The application of recycled concrete in building structures can not only effectively reduce the generation of construction waste and reduce the excessive dependence on natural aggregates but can also promote the sustainable use of resources and meet the national “double carbon” strategic requirements. This study investigates the effect of the recycled aggregate replacement ratio on the seismic performance of concrete-filled steel tube column–composite beam frames. Five finite element models were developed, considering varying recycled aggregate replacement ratios and the presence or absence of column-end stirrup-confined reinforcement. Dynamic response analyses were conducted. The results reveal that replacing natural aggregates with recycled aggregates reduces the stiffness of concrete-filled steel tube columns by weakening the core concrete, negatively impacting seismic performance and increasing structural stiffness damage. Column-end stirrup-confined reinforcement reduces interface slip between the core concrete and the steel tube by directly restraining the core concrete, thereby enhancing the bending stiffness of the concrete-filled steel tube column and improving the seismic performance of the structure. The seismic performance of recycled concrete frames with column-end stirrup-confined reinforcement is superior to that of conventional concrete frames, demonstrating that column-end reinforcement can effectively mitigate the adverse effects of recycled aggregate replacement on the structure’s seismic performance.

## 1. Introduction

Recycled concrete, produced by crushing, cleaning, and mixing waste concrete based on gradation, serves as a sustainable alternative to partially replace natural aggregates in conventional concrete [1]. With the rapid urbanization in China, the large volume of construction waste generated each year places immense pressure on the environment. The use of recycled concrete in building structures not only helps reduce construction waste pollution but also promotes sustainable resource use, aligning with the national “dual carbon” strategy [2].

Recent advancements in the study of the mechanical properties and durability of recycled concrete have been significant. Experimental analysis of specimens with varying recycled aggregate replacement ratios shows that the density, compressive strength, and toughness of recycled concrete decrease as the replacement ratio increases [3,4,5]. Research on the effect of recycled coarse aggregate replacement on the ductility and elastic modulus of recycled concrete indicates a reduction in both properties with higher replacement ratios; specifically, a 100% replacement ratio resulted in a 45% decrease in the elastic modulus [6,7]. Liu et al. [8] studied the shear behavior of recycled aggregate concrete, revealing that an increased replacement ratio led to a reduction in peak shear strain and greater brittleness. Additionally, Ding et al. [9] proposed a unified formula for the mechanical properties of recycled concrete, integrating experimental data across various strength grades and replacement ratios.

Regarding seismic performance, Choi et al. [10] and Ma et al. [11] conducted studies on the compressive strength of reinforced recycled concrete columns and found that ductility and energy dissipation decreased with higher replacement ratios. Studies by Tang et al. [12] and Yang et al. [13] on recycled aggregate concrete-filled steel tubular (RAC-FST) columns demonstrated that these columns maintain superior bearing and energy dissipation capacities compared to conventional concrete-filled steel tubular columns. Xiao et al. [14,15] performed shaking table and cyclic loading tests on recycled concrete frames and found that the ultimate bearing capacity of recycled concrete frames was reduced by 2.3% to 5.7% compared to conventional concrete frames, although recycled concrete frames still exhibited good bearing and deformation capacity. The research on the seismic performance of recycled concrete has been very sufficient. However, there is still a lack of research on the seismic performance of recycled concrete-filled steel tubular column–composite beam frame structures.

Concrete-filled steel tubular (CFST) structures have seen rapid development and are widely used in China. The CFST column–composite beam frame structure combines the strengths of both concrete and steel, significantly enhancing the seismic performance of the structure [16,17,18,19]. Structural reinforcement and ductility design are the key issues to improve the seismic performance of the structure. Laterza et al. [20] conducted bending moment–curvature analysis through OpenSees and proved that the special details of colored jade in the key areas of beams and columns can ensure sufficient curvature ductility. Among the existing reinforcement methods, the more common ones are welding steel plates or angle steel at the end of the column [21]; setting stiffeners, ribs, or tie rods [22,23,24]; and stainless steel–concrete–carbon steel double-skin tubular [25], carbon fiber-reinforced polymer (CFRP) [26], or fiber-reinforced polymer (FRP) wrapping [27,28,29]. Previous research by Ding et al. [30] investigated the axial compression and seismic performance of CFST columns, revealing that column-end stirrup-confined reinforcement reduces interface slip, directly restrains the core concrete, improves bending resistance, and enhances energy dissipation capacity. This reinforcement increases the bending stiffness of CFST columns by 10% and their bearing capacity by 20–50%. Xu et al. [2] found that the combination of column-end stirrup-confined reinforcement and girder heightening improves the ultimate seismic capacity of the structure, delays the emergence of a “compression hinge” and “tension hinge” at the column ends, and increases the seismic energy dissipation and anti-collapse capacity by 40%.

Building on this research, the present study further explores the seismic performance of recycled concrete-filled steel tubular column–composite beam frame structures. The main objectives of this study are as follows: (1) A finite element model of multi-group recycled concrete-filled square steel tubular column–composite beam frame structures is established, based on the constitutive relationship of recycled concrete at different replacement ratios proposed by Ding et al. [9]. The replacement ratio of recycled concrete aggregates is treated as the research variable, and the structural parameters are obtained to discuss the influence of recycled aggregate replacement on seismic performance. (2) The seismic response results of recycled concrete frames with column-end stirrup-confined reinforcement are compared with conventional concrete frames to assess the influence of column-end stirrup-confined reinforcement on the seismic performance of recycled concrete frames.

## 2. Finite Element Model

### 2.1. Overview of Example

**Figure 1:**
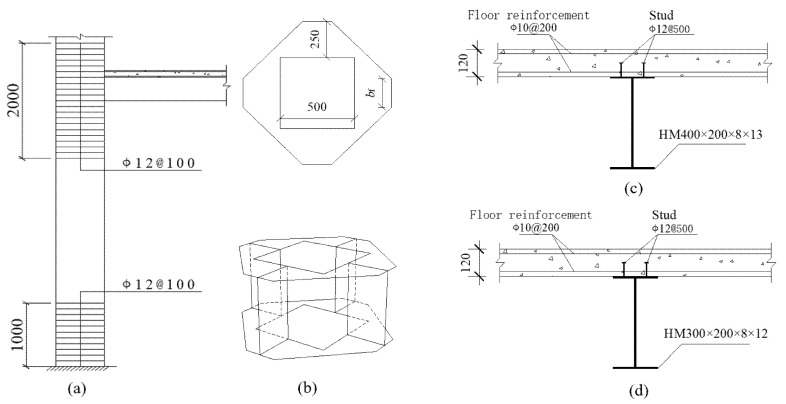
Schematic diagram of each section of the model example [2]: (**a**) arrangement range of column-end stirrup-confined reinforcement; (**b**) outer stiffening ring; (**c**) main beam; (**d**) secondary beam; unit: mm.

**Figure 2:**
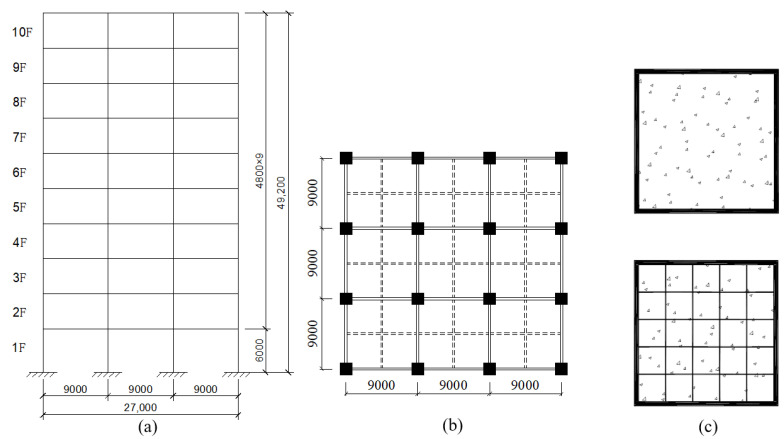
Diagram of CFST column–composite beam frame [2]: (**a**) structural facade diagram; (**b**) column grid layout; (**c**) arrangement of the column-end stirrup-confinement; unit: mm.

This study examines a 3 × 3 span CFST column–composite beam frame structure with a height of 49.2 m and a span of 9 m, as. The structure utilizes a concrete-filled square steel tubular (CFST) column with cross-sectional dimensions of 490 mm × 490 mm and a steel tube wall thickness of 5 mm. As shown in Figure 1a HRB400 tensile steel bars (Grade HRB400 steel) with a spacing of 100 mm are arranged in the range of 1000 mm at each joint of the bottom three layers. The main and secondary beams have dimensions of HM400 × 200 × 8 × 13 and HM300 × 200 × 8 × 12, respectively, where the first number refers to the height, the second to the width, the third to the thickness, and the fourth to the length. An outer stiffening ring is used between the beams and columns. Additionally, two rows of 19 studs (Φ19 at 500 mm spacing) are placed on the upper flange of the steel beam, serving as shear connectors with the concrete floor. The concrete floor slab is 120 mm thick, with two layers of Φ10 at 200 mm spacing steel bars arranged in both directions. The thickness of the protective layer is 25 mm. According to whether the column-end stirrup-confined reinforcement is applied and different recycled aggregate replacement rates, this paper establishes 5 models, as shown in Table 1. Figure 1 shows the schematic diagram of the main sections of the model, while the overview of the calculation examples and the schematic diagram of the composite frame are presented in Table 1 and Figure 2, respectively.

In each example, the influence of the replacement rate on the seismic performance of the structure is discussed by using 50% and 100% replacement rates. The strength grade of both conventional concrete and recycled aggregate concrete (at various replacement ratios) is C50, while the strength grade of the steel is Q235. The column-end stirrup-confined reinforcement and the floor reinforcement mesh use HRB400-grade steel bars. Steel beams and the floor are connected using ML15-grade studs, according to the relevant specifications. Based on the previous research results of the subject group [9], the elastic parameters of various materials, including concrete, steel tubes, steel beams, steel mesh, and studs, are presented in Table 2.

### 2.2. Finite Element Model Establishment

**Figure 3:**
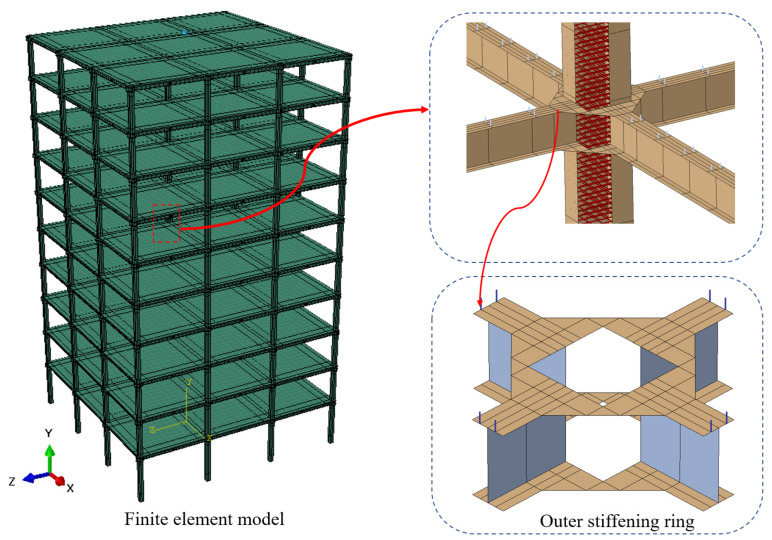
Finite element model of spatial frame.

In this paper, the research group used the nonlinear finite element software ABAQUS 2020 to conduct numerical simulations. The example frame consists primarily of 13 components, including the steel tube, concrete column, main beam, secondary beam, outer stiffening ring, concrete floor, steel mesh, and so on. The finite element model is illustrated in Figure 3. During the meshing process, refined grids are used for the beam–column joints and the joints between the floor and the studs, while the other components are meshed with a general grid. The specific element types used for each component are as follows: C3D8R elements are applied to the core concrete column and the concrete slab. S4R elements are used for the outer stiffening ring and the steel tube column, considering the neglect of stress in the thickness direction of the steel tube. B31 elements are employed for the studs. T3D2 elements are used for the column-end stirrup confinement and the steel bars embedded in the slab [30].

In the interaction module, as detailed in Reference [31], a “surface-to-surface” constraint is defined between the surface of the core concrete column and the inner surface of the steel tube column. The surface of the core concrete column is designated as the master surface, while the inner surface of the steel tube column serves as the slave surface. Steel bars in the concrete slabs and studs are embedded in the floor, with these steel bars or studs treated as the built-in area. The floor slabs are considered the host area. The stirrups at the column ends are treated in the same manner as the steel bars in the concrete slabs and studs, and a “binding constraint” is applied between the steel tube column and the outer stiffening ring.

### 2.3. Material Constitutive Models

The plastic-damage constitutive parameters for both conventional concrete and recycled concrete are defined using the theory of concrete damage-specific strength [32]. These parameters include a dilation angle of 40°, an eccentricity of 0.1, a ratio of the initial equibiaxial compressive yield stress to the initial uniaxial compressive yield stress of 1.331, a ratio of the second stress invariant on the tensile meridian to that on the compressive meridian of 0.64, and a viscosity coefficient of 0.0005.

Considering the restraint effect of the steel tube on the concrete, the descending branch of the stress–strain curve is adjusted accordingly. Both conventional and recycled concrete follow the plastic-damage model proposed by Ding et al. [32]. The dimensionless stress–strain curve equation is as follows:

(1) $$y=\left\{\begin{array}{cc} \frac{A_{i}x+(B_{i}-1)x^{2}}{1+(A_{i}-2)x+B_{i}x^{2}} & x\leq 1\\ \frac{x}{\alpha_{i}{\left(x-1\right)}^{2}+x} & x>1 \end{array}\right.$$

where $A_{i}$ represents the ratio of the elastic modulus to the peak secant modulus of concrete; $B_{i}$ denotes the attenuation degree of the elastic modulus on the ascending curve of concrete; $\alpha_{i}$ is the descending branch parameter; $\alpha_{1}$ = 4.0 × 10^−3^ *f*_cu_^1.5^ when the transverse stirrup ratio or steel ratio is 0; and $\alpha_{1}$ = 0.15 when the transverse stirrup ratio or steel ratio is greater than 2%. When the transverse stirrup ratio or steel ratio is between 0 and 2%, the linear interpolation value is calculated according to the steel ratio of the concrete-filled steel tube column.

When *i* = 1, *y* = *σ*/*f*_c_ and *x* = *ε*/*ε*_c_, where *f*_c_ is the uniaxial compressive strength, and *ε*_c_ is the uniaxial compressive peak strain; when *i* = 2, *y* = *σ*/*f*_t_ and *x* = *ε*/*ε*_t_, where *f*_t_ is the uniaxial tensile strength, and *ε*_t_ is the uniaxial tensile peak strain. The expressions of some specific parameters are shown in Table 3.

The elastic–plastic hybrid strengthening model proposed by Zhang et al. [33] is used for modeling the steel tube, steel beams, outer stiffening ring, steel bars in the concrete slabs, and stirrups at the column ends. The specific parameters are as follows: the yield stress at zero plastic strain is taken as the yield strength of steel *f*_y_, the kinematic hardening parameter C1 is taken as 7500, the maximum change of yield surface Q- is taken as 0.5 *f*_y_, the change ratio of back stress γ is taken as 50, and the hardening parameter b is taken as 0.1.

A combined hardening model, considering the ductile damage of steel induced by large plastic deformation, is adopted for the steel components. The fracture strain and stiffness damage factor are calculated using the following formulas [34]:

(2) $$\varepsilon_{f}=7.72f_{y}^{-0.4}$$

(3) $$D_{s}=0.96{\left({\bar{\mu }}^{\mathrm{pl}}/{\bar{\mu }}_{f}\right)}^{2.42}$$

In these formulas, *ε*_f_ is the fracture strain; *f*_y_ is the yield strength of steel; *D*_s_ is the stiffness damage factor; and $\bar{\mu }$^pl^ and $\bar{\mu }$_f_ are the plastic and ultimate displacements of steel during tension, respectively.

An elastic–plastic constitutive model with isotropic hardening is applied to the studs, and the stress–strain relationship is given by [34]:

(4) $$\sigma =\left\{\begin{array}{cc} E_{s}\varepsilon & \varepsilon \leq \varepsilon_{y}\\ f_{y}+0.01E_{s}(\varepsilon -\varepsilon_{y}) & \varepsilon_{y}<\varepsilon \leq \varepsilon_{u}\\ 1.2f_{y} & \varepsilon >\varepsilon_{u} \end{array}\right.$$

In the formula, *σ* is the stress; *E*_s_ = 2.06 × 10^5^ MPa is the elastic modulus; *ε* is the strain; *f*_y_ is the yield strength; *ε*_y_ is the yield strain; and *ε*_u_ is the ultimate strain, which is taken as *ε*_u_ = 21*ε*_y_.

### 2.4. Boundary Conditions and Seismic Wave Input

In ABAQUS finite element software, a fully fixed constraint is applied at the bottom of the spatial frame model columns to limit displacement and rotation in the X, Y, and Z directions (i.e., U1 = U2 = U3 = UR1 = UR2 = UR3 = 0).

**Figure 4:**
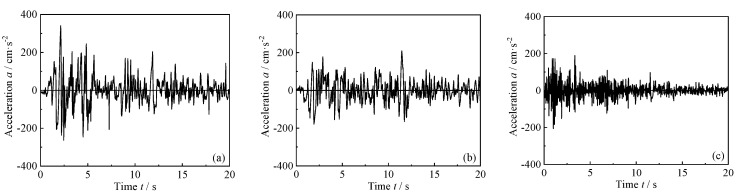
Selected seismic waves: (**a**) N–S direction; (**b**) E–W direction; (**c**) U–D direction.

The El Centro wave front with a duration of 20 s is selected for the structural dynamic response analysis. The original acceleration data in three directions are shown in Figure 4. According to GB 50009-2012 “Code for Seismic Design of Buildings” [35], the peak acceleration of the seismic wave is modulated to values of 0.14 g, 0.4 g, 0.62 g, 0.85 g, 1.0 g, 1.25 g, 1.5 g, 1.75 g, and 2.0 g, respectively. When applying the three-dimensional seismic wave for seismic response analysis, the acceleration is also modulated in each direction according to the ratio of 1:0.85:0.65.

Two analysis steps are used in ABAQUS: In the first step, gravity loads and out-of-floor loads are applied. The roof live load, floor live load, and infill walls load are converted into mass density and evenly distributed across the response load area. Gravity acceleration is applied along the *Y*-axis. In the second step, seismic loads are applied, with the original seismic wave data amplitude modulated to the desired levels and applied across the entire model for seismic response analysis.

### 2.5. Frequency and Vibration Mode Analysis

In ABAQUS, a linear perturbation analysis step is set, using the Lanczos method for modal analysis of the five examples listed in Table 1. The effects of different recycled aggregate replacement ratios and column-end stirrup confinement on the natural frequency of the structure are shown in Table 4 and Table 5, respectively. The following conclusions can be drawn based on the natural frequency: (1) The replacement of recycled aggregate reduces the natural frequency of the composite frame. As the replacement ratio of recycled aggregate increases, the natural frequency decreases more significantly. A 50% replacement ratio reduces the natural frequency by 2.15%, and a 100% replacement ratio reduces it by 4.72%. (2) Column-end stirrup-confined reinforcement significantly increases the natural frequency of the composite frame. Compared to the reference frame KJ-1, the first-order natural frequency of KJ-4 (*η*_r_ = 0.5) and KJ-5 (*η*_r_ = 1) increases by 6.87% and 4.29%, respectively. (3) Recycled aggregate replacement and column-end stirrup confinement have no significant effect on the vibration modes of the model cases.

## 3. Structural Response Analysis

### 3.1. Ultimate Seismic Capacity and Interlayer Displacement Angle

The finite element analysis results indicate that under the influence of one-dimensional seismic waves, the peak ground acceleration (PGA) that the frames KJ-1 and KJ-2 (*η*_r_ = 0.5) can withstand is 1.5 g. However, considering the excessive interlayer displacement angle of frame KJ-3 (*η*_r_ = 1) under a 1.5 g horizontal seismic wave, it was determined that the maximum PGA that frame KJ-3 can withstand is 1.25 g. Frames KJ-4 (*η*_r_ = 0.5) and KJ-5 (*η*_r_ = 1), which incorporate column-end stirrup-confined reinforcement, can withstand a PGA of up to 1.75 g. Under the action of three-dimensional seismic waves, the PGA that frames KJ-1, KJ-2, KJ-3, and KJ-5 can withstand is 0.62 g, while frame KJ-4 can withstand up to 0.85 g.

**Figure 5:**
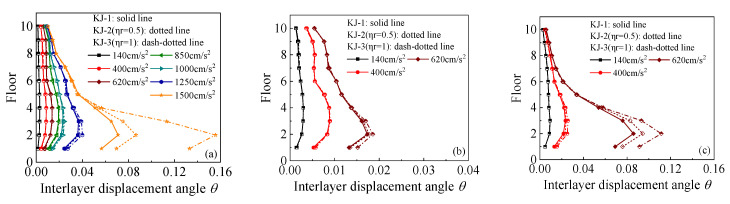
Influence of different recycled aggregate replacement ratios on interlayer displacement angles: (**a**) N–S seismic waves; (**b**) X direction of tri-directional earthquake; (**c**) Z direction of tri-directional earthquake.

**Figure 6:**
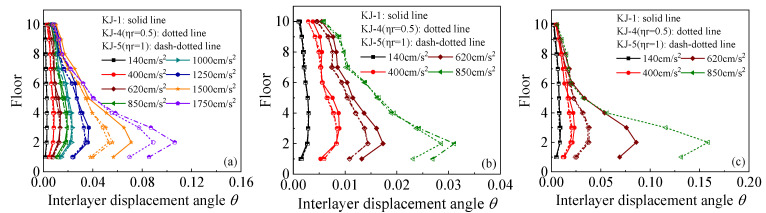
Influence of column-end stirrup-confined reinforcement on interlayer displacement angles: (**a**) N–S seismic waves; (**b**) X direction of tri-directional earthquake; (**c**) Z direction of tri-directional earthquake.

The interlayer displacement angle responses for each model under various seismic conditions are shown in Figure 5 and Figure 6. Figure 5 illustrates the influence of the recycled aggregate replacement ratio on the interlayer displacement angle of the frame, while Figure 6 compares the interlayer displacement angles of the reference frame and the column-end stirrup-confined frame for the two replacement ratios. This comparison highlights the effect of column-end stirrup-confined reinforcement on the interlayer displacement angle. Key observations from the diagrams include the following: (1) As the intensity of the seismic wave increases, the interlayer displacement angle of the model also increases, with the maximum displacement occurring at the second layer. Once the structure enters the elastic–plastic stage, the changes in the interlayer displacement angle become more pronounced. (2) As the recycled aggregate replacement ratio increases, the interlayer displacement angle of the frame also increases. This is particularly evident under the action of a 1.5 g horizontal seismic wave in the one-dimensional seismic wave case and under the action of a 0.62 g seismic wave in the three-dimensional seismic wave case. (3) The interlayer displacement angles of frames KJ-4 and KJ-5 are notably lower than those of the reference frame (KJ-1), indicating that the column-end stirrup-confined reinforcement effectively mitigates the negative impact of the recycled aggregate replacement on the interlayer displacement angle of the structure.

### 3.2. Maximum Lateral Displacements

**Figure 7:**
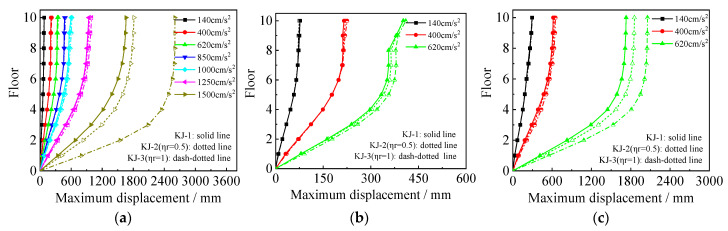
Influence of different replacement ratios of recycled aggregate on maximum floor displacements: (**a**) N–S seismic waves; (**b**) X direction of tri-directional earthquake; (**c**) Z direction of tri-directional earthquake.

**Figure 8:**
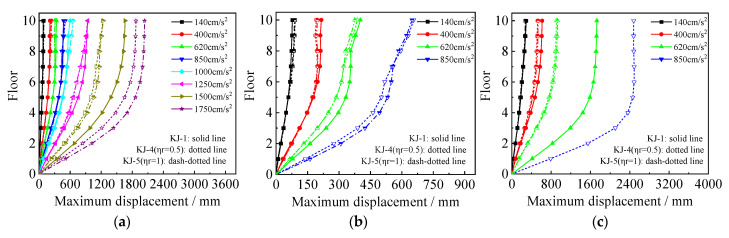
Influence of column-end stirrup-confined reinforcement on maximum floor displacements: (**a**) N–S seismic waves; (**b**) X direction of tri-directional earthquake; (**c**) Z direction of tri-directional earthquake.

The maximum floor displacements under one-dimensional and three-dimensional seismic waves for each model are shown in Figure 7 and Figure 8, respectively. The following observations can be drawn from the figures: (1) Under the influence of one-dimensional seismic waves with an intensity of 1.5 g, the displacement in frame KJ-3 is excessively large, indicating that the peak ground acceleration (PGA) that frame KJ-3 can withstand is 1.25 g. For the three-dimensional seismic waves, the maximum floor displacements of the recycled concrete frames increase by 7.29% and 18.85%, respectively, compared to the conventional concrete frame for the two replacement ratios. This indicates that the higher the replacement rate of recycled aggregate, the lower the elastic modulus of concrete, resulting in larger maximum floor displacements of the structure. (2) Focusing on the maximum floor displacements of frames KJ-1, KJ-4, and KJ-5 under a horizontal PGA of 1.5 g, it is observed that the maximum floor displacements of the recycled concrete frames with column-end stirrup-confined reinforcement are reduced by 25.9% and 25.4%, respectively, compared with the reference frame KJ-1. This demonstrates the significant constraint effect of column-end stirrup-confined reinforcement on floor displacements, effectively offsetting the adverse impact of recycled aggregate replacement on structural displacements.

### 3.3. Maximum Interlayer Shear Force and Overturning Moment

**Figure 9:**
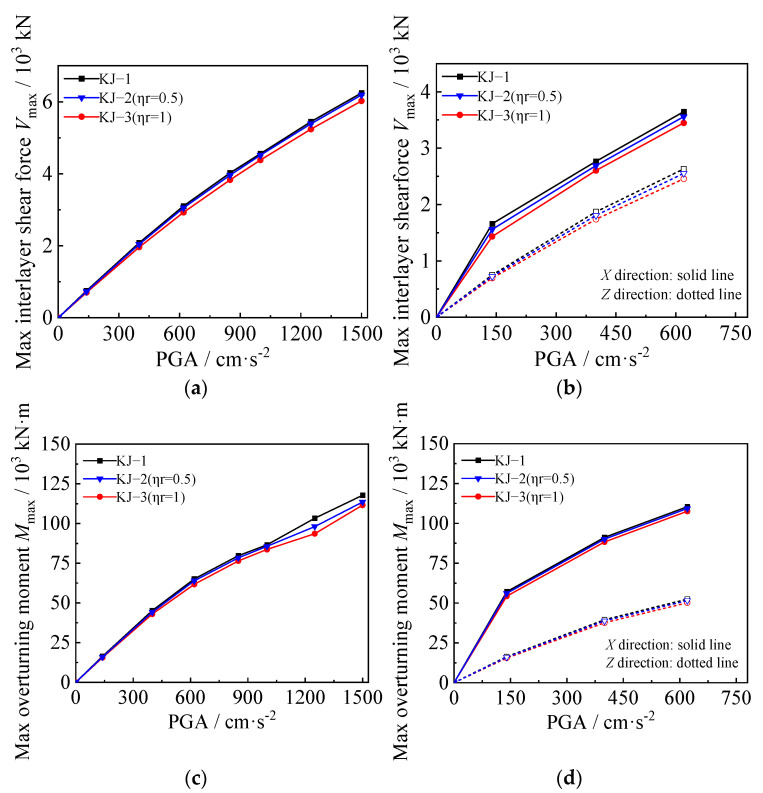
Influence of different replacement ratios of recycled aggregate on maximum interlayer shear force and overturning moment: (**a**,**c**) unidirectional horizontal earthquake; (**b**,**d**) tri-directional earthquake.

**Figure 10:**
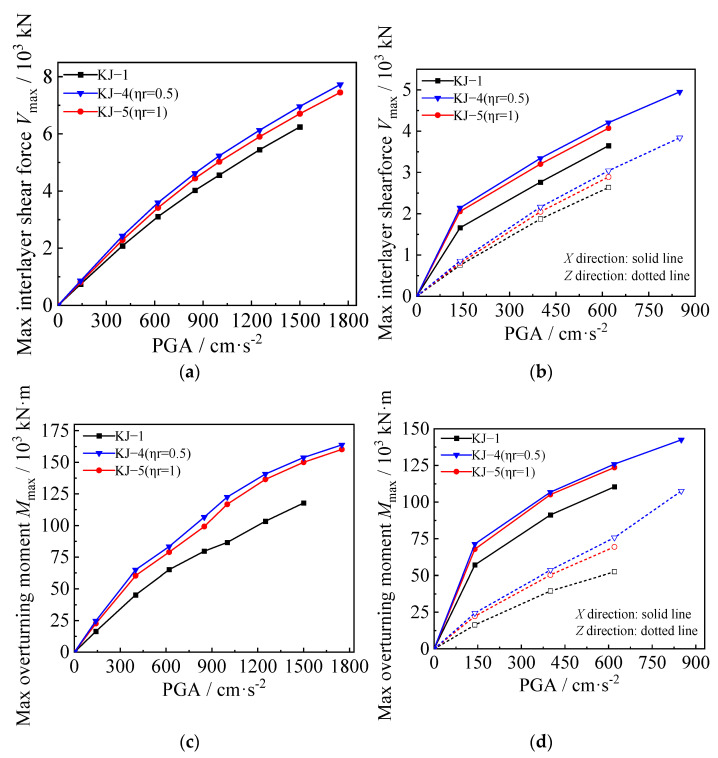
Influence of column-end stirrup-confined reinforcement on maximum interlayer shear force and overturning moment: (**a**,**c**) unidirectional horizontal earthquake; (**b**,**d**) tri-directional earthquake.

The maximum interlayer shear force and overturning moment under one-dimensional and three-dimensional seismic waves for each model are shown in Figure 9 and Figure 10, respectively. The following observations can be drawn from the figures: (1) When the seismic wave intensity is low, the maximum interlayer shear force and overturning moment of the composite frame increase rapidly with the increase of seismic wave intensity. When the seismic wave intensity reaches a high level, the structure enters the elastic–plastic stage, and its growth rate is significantly slowed down. It shows certain nonlinear characteristics. (2) Under the influence of one-dimensional seismic waves, the maximum interlayer shear force and overturning moment of the structure are reduced by 3.5% and 9.4%, respectively. Under the action of three-dimensional seismic waves, the maximum interlayer shear force and overturning moment of the structure are reduced by 3% and 2.5%, respectively. It can be seen that when the replacement ratio of recycled aggregate is low, the interlayer shear force and overturning moment of the structure do not change significantly. With a further increase in the replacement ratio, the reduction in interlayer shear force and overturning moment will increase significantly. (3) Combined with the influence of recycled aggregate replacement on the interlayer displacement angle and displacement envelope of the structure, the replacement of recycled aggregate will cause the structure to bear a smaller shear force and overturning moment while generating a larger interlayer displacement, which shows that the replacement of recycled aggregate is unfavorable to the seismic resistance of the structure. (4) Column-end stirrup-confined can significantly improve the stiffness of the CFST column and bear a greater interlayer shear force and bending moment while reducing the interlayer displacement of the structure. As shown in Figure 9c,d and Figure 10c,d, compared with the reference frame, the interlayer shear force of the recycled concrete frames with column-end stirrup-confined reinforcement under the two replacement ratios is increased by 10–20%, and the maximum overturning moment is increased by 30–40%, indicating that column-end stirrup-confined reinforcement can effectively compensate for the adverse effects of recycled aggregate replacement on the lateral stiffness of CFST columns.

### 3.4. Stress–Strain Curve and Interface Slip

The stress–strain curves and interface slip at the column end are analyzed based on the calculation results, when the horizontal peak ground acceleration (PGA) is 1 g. The influence of the recycled aggregate replacement ratio and column-end stirrup-confined reinforcement is explored.

**Figure 11:**
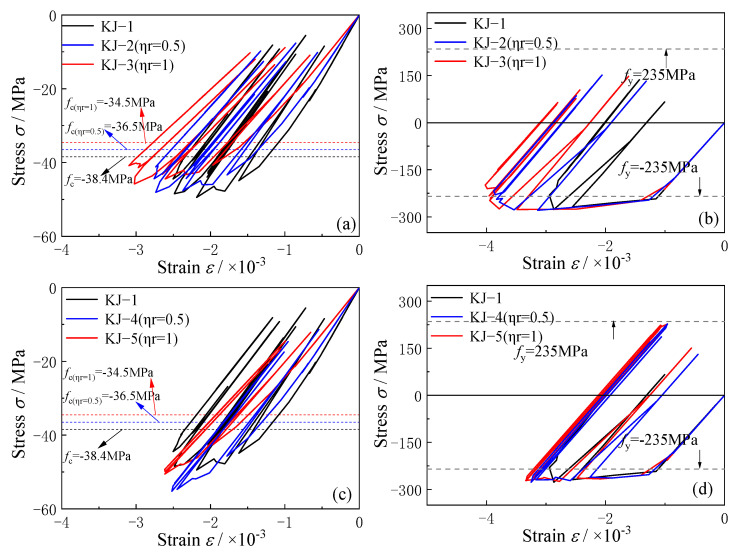
Stress–strain curves of CFST columns: (**a**,**c**) concrete at the bottom of the central column; (**b**,**d**) steel tube at the bottom of the central column.

The stress–strain curves for the core concrete and steel tube column at the bottom of the column for each frame are shown in Figure 11. Key observations include the following: (1) The replacement of recycled aggregate increases the strain in the core concrete, reduces the stress level, and increases the strain in the steel tube. Compared with the reference frame KJ-1, the maximum strain of the core concrete in KJ-2 (*η*_r_ = 0.5) and KJ-3 (*η*_r_ = 1) increases by 10.2% and 23.7%, respectively. In addition, the strain in the longitudinal steel tube rises by 31.7% and 36%, respectively. This indicates that recycled aggregate replacement reduces the strength of the core concrete and the stress level in the core, weakening the resistance of the steel tube. As the replacement ratio of recycled aggregate increases, the resistance of CFST columns decreases further. (2) Figure 11c,d show that column-end stirrup-confined reinforcement improves the stress level by directly restraining the core concrete. As a result, the stress at the column end of the reinforced frames KJ-4 and KJ-5 increases, and the resistance of the steel tube is enhanced. This demonstrates that column-end stirrup-confined reinforcement can effectively offset the adverse effects of recycled aggregate replacement on the resistance of concrete-filled steel tubular (CFST) columns.

**Figure 12:**
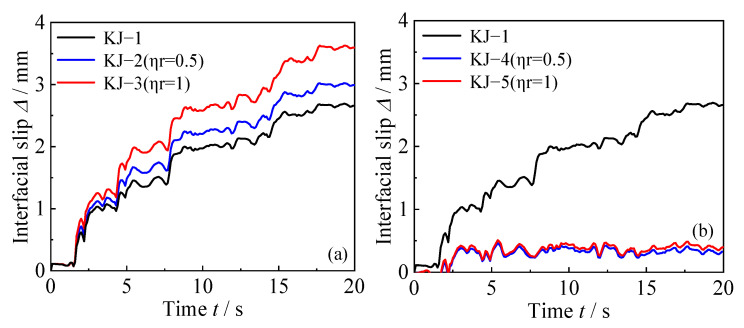
Time history curve of longitudinal displacement difference of CFST columns: (**a**) influence of different replacement ratios of recycled aggregate; (**b**) influence of column-end stirrup-confined reinforcement.

In order to prevent the influence of the column-end stirrup-confined reinforcement region on the results, the point of contraflexure is selected for analysis. The longitudinal displacement difference between the steel tube and concrete at a height of 2000 mm at the bottom of the side column is analyzed, as shown in Figure 12. The following conclusions can be drawn: (1) Compared with the reference frame KJ-1, the interface slip in the recycled concrete frame increases by 12.9% and 35.5% for the two replacement ratios, respectively. This indicates that as the recycled aggregate replacement ratio increases, the interface slip at the column end also increases. (2) In contrast, the interface slip of the reinforced frames KJ-4 and KJ-5 is significantly reduced compared to the reference frame KJ-1. This demonstrates that column-end stirrup-confined reinforcement can effectively mitigate the adverse effects of recycled aggregate replacement on interface slip by directly restraining the core concrete. (3) In the frame without column-end stirrup-confined reinforcement, the interface slip continues to increase over time, while in the frame with column-end stirrup-confined reinforcement, the interface slip remains more stable, as shown by the time history curve.

### 3.5. Axial Compression Ratio Time History Curve

**Figure 13:**
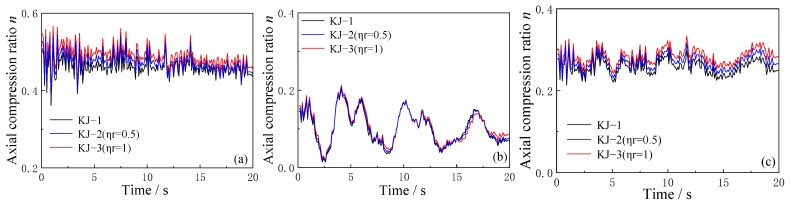
Influence of different replacement ratios of recycled aggregate on time history curves of axial compression ratio: (**a**) central column; (**b**) corner column; (**c**) side column.

**Figure 14:**
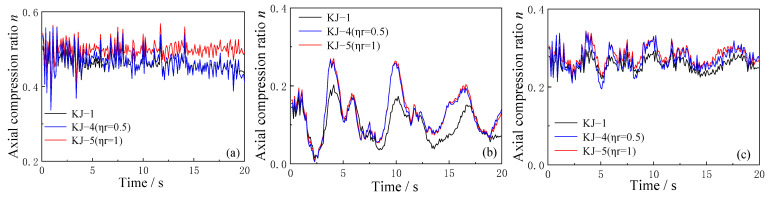
Influence of column-end stirrup-confined reinforcement on time history curves of axial compression ratio: (**a**) central column; (**b**) corner column; (**c**) side column.

The effect of the recycled aggregate replacement ratio and column-end stirrup-confined reinforcement on the axial compression ratio over time is analyzed using the model in which the PGA of the three-dimensional seismic wave is 0.62 g. The results are shown in Figure 13 and Figure 14.

As shown in Figure 13, the replacement of recycled aggregate increases the axial compression ratio at the end of the structural column. The higher the replacement ratio, the more pronounced the increase in the axial compression ratio. This suggests that recycled aggregate replacement leads to higher axial compression at the column ends, potentially influencing the overall performance of the structure under seismic loads.

Comparing the axial compression ratio time history curves for frames KJ-1, KJ-4, and KJ-5 in Figure 14, it is evident that column-end stirrup-confined reinforcement does not significantly affect the axial compression ratio at the structural column ends. However, the axial compression ratio of the recycled concrete frame with column-end stirrup-confined reinforcement remains higher than that of the conventional concrete frame without reinforcement. This indicates that while column-end stirrup-confined reinforcement may not directly affect the axial compression ratio, it still plays a role in maintaining higher structural integrity under seismic forces.

## 4. Structural Plastic Energy Dissipation and Stiffness Damage

### 4.1. Structural Plastic Energy Dissipation

**Figure 15:**
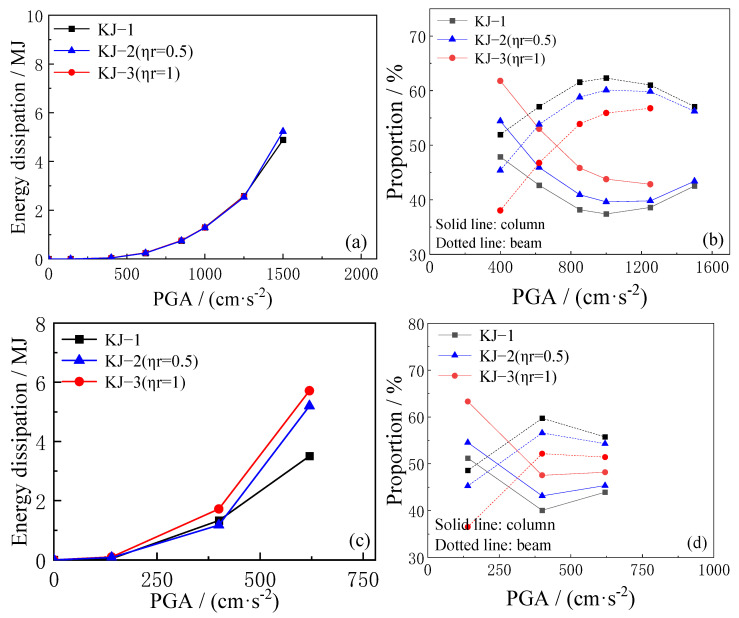
Influence of different replacement ratios of recycled aggregate on plastic energy dissipation. (**a**,**b**) unidirectional horizontal earthquake; (**c**,**d**) tri-directional earthquake.

**Figure 16:**
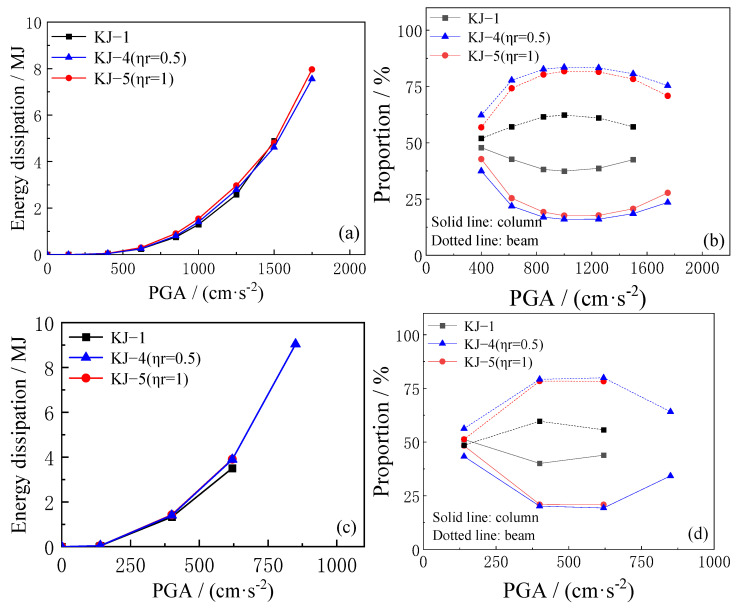
Influence of column-end stirrup-confined on plastic energy dissipation. (**a**,**b**) unidirectional horizontal earthquake; (**c**,**d**) tri-directional earthquake.

The total plastic energy consumption of the structure and the proportion of energy consumption of beams and columns under different seismic intensities are analyzed based on the finite element calculation results. The findings are illustrated in Figure 15 and Figure 16. The key observations are as follows: (1) When the structure does not enter the elastoplastic stage under the action of small seismic waves, the total plastic energy dissipation remains low. At this stage, the replacement ratio of recycled aggregate has minimal effect on the total plastic energy dissipation. However, as the seismic wave intensity increases, the structure’s plastic energy dissipation increases. Additionally, the higher the recycled aggregate replacement ratio, the greater the total plastic energy dissipation. (2) Recycled aggregate replacement significantly reduces the seismic capacity of CFST columns while simultaneously increasing the proportion of plastic energy dissipation in the CFST column. As the recycled aggregate replacement ratio increases, the proportion of energy dissipation in the CFST column also increases. (3) Compared to the reference frame KJ-1, under the same earthquake intensity, the total plastic energy dissipation of the reinforced frames KJ-4 (*η*_r_ = 0.5) and KJ-5 (*η*_r_ = 1) is higher, with a greater proportion of energy dissipation occurring in the beam. This indicates that column-end stirrup-confined reinforcement can effectively mitigate the reduction in resistance of CFST columns due to recycled aggregate replacement, allowing the structure to maintain the “strong column and weak beam” energy dissipation mechanism.

### 4.2. Development of Structural Plastic Hinge

**Figure 17:**
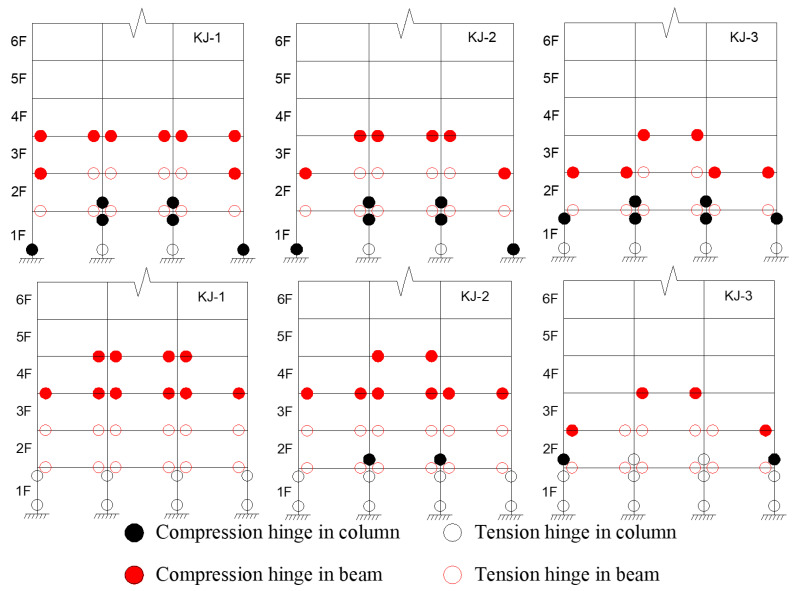
Influence of different replacement ratios of recycled aggregate on the distribution of plastic hinges.

**Figure 18:**
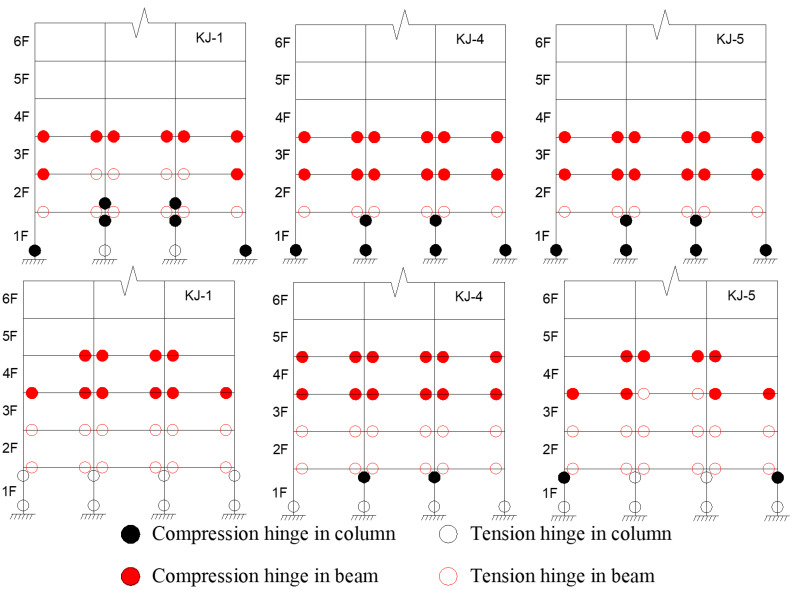
Influence of column-end stirrup confinement on the distribution of plastic hinges.

The criteria for determining the plastic hinge in the structure, based on previous research [2], are as follows: when the compressive yield of the bottom flange of the beam end reaches 1/3 of the height of the section, it is the compression hinge at the beam end; when the tensile yield of the bottom flange of the beam end reaches 1/3 of the height of the section, it is the tension hinge at the beam end. When the compression yield of the steel tube section at the column end reaches 1/3 of the section height, it is the compression hinge at the column end; when the tensile yield of the steel tube section at the column end reaches 1/3 of the section height, it is the tension hinge at the column end. Based on these criteria and the finite element calculation results, the distribution of plastic hinges for the model under the influence of one-dimensional and three-dimensional seismic waves is shown in Figure 17 and Figure 18. Key observations include the following: (1) Recycled aggregate replacement negatively affects the seismic resistance of the structural column ends. It accelerates the formation of the plastic hinge at the column end and shortens the transition from the “compression hinge” to the “tension hinge”. An increase in the recycled aggregate replacement ratio also leads to a higher number of plastic hinges at the column end, while the number of plastic hinges at the beam end decreases. (2) Compared to the reference frame KJ-1, frames KJ-4 and KJ-5, which include column-end stirrup-confined reinforcement, show fewer plastic hinges at the column end and more plastic hinges at the beam end. This suggests that column-end stirrup-confined reinforcement enhances the resistance of the column end, effectively mitigating the adverse effects of recycled aggregate replacement on the development of plastic hinges.

### 4.3. Stiffness Damage

**Figure 19:**
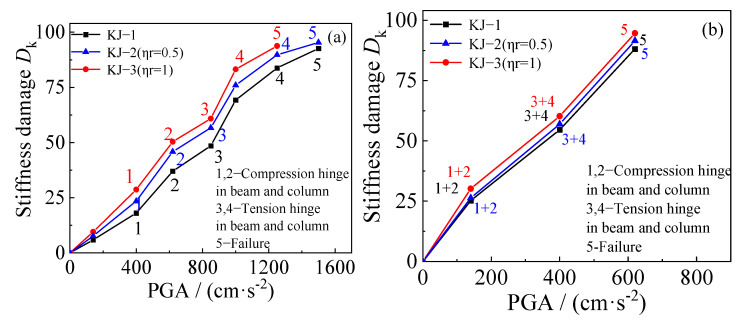
Influence of different replacement rates of recycled aggregate: (**a**) unidirectional horizontal earthquake; (**b**) tri-directional earthquake.

**Figure 20:**
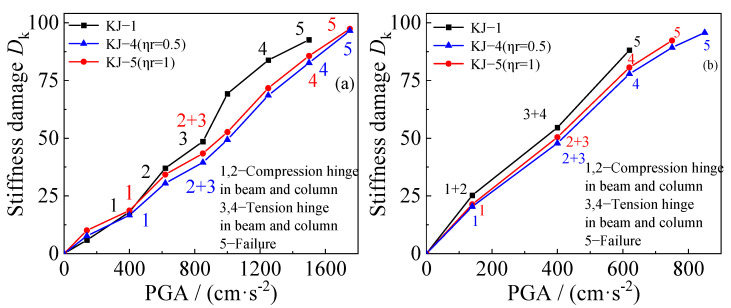
Influence of column-end stirrup-confined on stiffness damage: (**a**) unidirectional horizontal earthquake; (**b**) tri-directional earthquake.

The diagrams showing the change in stiffness damage for each example under different seismic waves are compared based on the finite element results, as shown in Figure 19 and Figure 20. The following observations can be made: (1) Recycled aggregate replacement does not significantly alter the structural failure mode. In all examples, the failure mode follows the “strong column and weak beam” pattern, where failure at the beam end hinge leads to structural collapse. (2) As the recycled aggregate replacement ratio increases, the collapse resistance of the structure weakens. Consequently, the rate of structural stiffness damage increases, and the overall stiffness damage becomes more pronounced. (3) A comparison of the stiffness damage results for the benchmark frame KJ-1 and the reinforced frames KJ-4 (*η*_r_ = 0.5) and KJ-5 (*η*_r_ = 1) reveals that column-end stirrup-confined reinforcement improves structural stiffness. The stiffness damage in frames KJ-4 and KJ-5 is lower than in the reference frame KJ-1. This indicates that column-end stirrup-confined reinforcement enhances the stiffness of the structural column end, improving the seismic performance of the structure. In other words, the reinforcement effectively mitigates the adverse effects of recycled aggregate replacement on structural stiffness damage.

## 5. Conclusions

This paper investigates the impact of the recycled aggregate replacement ratio and column-end stirrup-confined reinforcement on the seismic performance of CFST column–composite beam space frame structures under different seismic wave conditions. The study evaluates multiple aspects of the structure, including the interlayer displacement angle response, stress–strain behavior at the bottom of the middle column, interface slip between the core concrete and the steel tube, axial compression ratio time history, plastic energy dissipation, plastic hinge development, and stiffness damage. The key conclusions are as follows:

(1) Recycled aggregate replacement leads to a decrease in the structure’s natural frequency, an increase in the interlayer displacement angle, a decrease in the interlayer shear force and bending moment that the structure is subjected to, more interface slip between the core concrete and the steel tube, reduced plastic energy dissipation, and increased stiffness damage. The higher the recycled aggregate replacement ratio, the more significant the effect on seismic performance.
(2) Column-end stirrup-confined reinforcement reduces interface slip between the concrete column and the steel tube by directly restraining the core concrete. It enhances the energy dissipation capacity of the CFST column, increases the total plastic energy dissipation of the structure, and reduces the energy dissipation ratio of the CFST column. Additionally, it increases the number of plastic hinges at the beam end and reduces the number of plastic hinges at the column end, delaying the formation of a “compression hinge” and extending the transition to a “tension hinge”.
(3) The stirrup-confined recycled concrete frame exhibits a higher natural frequency, smaller interlayer displacement angle, lower stiffness damage, and better overall seismic performance than conventional concrete frames. This demonstrates that column-end stirrup-confined reinforcement can effectively mitigate the adverse effects of recycled aggregate replacement on seismic performance.
(4) Although recycled concrete exhibits lower material and mechanical properties compared to conventional concrete, its seismic performance can be significantly enhanced sometimes even surpassing that of traditional composite frames through effective column-end stirrup reinforcement. This suggests that recycled concrete is a promising alter-native for conventional concrete in seismic applications. However, the seismic performance of recycled concrete structures is influenced by factors such as the quality of recycled aggregates, construction processes, and reinforcement design. Future research should focus on optimizing the use of different recycled aggregate ratios, improving construction quality control, and refining reinforcement strategies. These efforts will provide crucial insights to ensure the practical application of recycled concrete in real-world engineering projects.

## Figures and Tables

**Table 1:** Overview of calculation examples.

Model Case	Column-End Stirrup Confined	Seismic Wave Direction	Recycled Aggregate Replacement Ratio/%
KJ-1	No	N–S; N–S, E–W, U–D	0
KJ-2	No	N–S; N–S, E–W, U–D	50
KJ-3	No	N–S; N–S, E–W, U–D	100
KJ-4	Yes	N–S; N–S, E–W, U–D	50
KJ-5	Yes	N–S; N–S, E–W, U–D	100

**Table 2:** Elastic parameters of various materials.

Material	Recycled Aggregate Replacement Ratio/%	Elastic Modulus/MPa	Poisson’s Ratio	Strength Grade
Concrete	0	34,998	0.2	C50
Concrete	50	29,748	0.2	C50
Concrete	100	24,498	0.2	C50
Steel bar		2.06 × 10^5^	0.285	HRB400
Stud		2.06 × 10^5^	0.285	ML15
Steel tube		2.06 × 10^5^	0.285	Q235
Steel beam		2.06 × 10^5^	0.285	Q235

**Table 3:** Expressions of parameter in concrete uniaxial stress–strain curve.

Force Mode	Compression	Tension
Conventional concrete	*f*_c_ = 0.4*f*_cu_^7/6^, *E*_c_ = 9500*f*_cu_^1/3^, *ε*_c_ = 291*f*_cu_^7/15^ × 10^−6^; *A*_1_ = 6.9*f*_cu_^−11/30^, *B*_1_ = 1.67(*A*_1_ − 1)^2^;	*f*_t_ = 0.24*f*_cu_^2/3^, *ε*_t_ = 33*f*_cu_^1/3^ × 10^−6^, *A*_2_ = 1.3, *B*_2_ = 0.15, *α*_2_ = 0.8
Recycled aggregate concrete	*f*_c_ = 0.4*f*_cu_^7/6^(1 − 0.1*η*_r_), *E*_c_ = 9500*f*_cu_^1/3^(1 − 0.3*η*_r_), *ε*_c_ = (1 + 0.2*η*_r_)291*f*_cu_^7/15^ × 10^−6^, *A*_1_ = 6.9*f*_cu_^−11/30^(1 − 0.3*η*_r_)(1 + 0.2*η*_r_)/(1 − 0.1*η*_r_), *B*_1_ = 1.67(*A*_1_ − 1)^2^	*f*_t_ = 0.24*f*_cu_^2/3^(1 − 0.1*η*_r_), *ε*_t_ = 33*f*_cu_^1/3^ × 10^−6^(1 + 0.1*η*_r_), *A*_2_ = 1.3(1 − 0.3*η*_r_)(1 + 0.1*η*_r_)/(1 − 0.1*η*_r_), *B*_2_ = 1.67(*A*_2_ − 1)^2^, *α*_2_ = 0.8

Note: *η*_r_ represents the replacement ratio of recycled aggregate.

**Table 4:** The influence of different replacement ratios of recycled aggregate on the structural natural vibration frequency.

Model No.	KJ-1/Hz	KJ-2 (*η*_r_ = 0.5)/Hz	Rate of Decrease/%	KJ-3 (*η*_r_ = 1)/Hz	Rate of Decrease	Vibration Modes
1	0.233	0.228	2.15	0.222	4.72%	Translational vibration
2	0.258	0.252	2.33	0.244	5.43%	Translational vibration
3	0.299	0.291	2.68	0.283	5.35%	Torsional vibration
4	0.735	0.717	2.48	0.696	5.31%	Bending vibration
5	0.807	0.785	2.73	0.76	5.82%	Bending vibration
6	0.936	0.91	2.78	0.881	5.88%	Torsional vibration

**Table 5:** The influence of column-end stirrup-confined reinforcement on the structural natural vibration frequency.

Model No.	KJ-1/Hz	KJ-4 (*η*_r_ = 0.5)/Hz	Rate of Increase/%	KJ-3 (*η*_r_ = 1)/Hz	Rate of Increase	Vibration Modes
1	0.233	0.249	6.87%	0.243	4.29%	Translational vibration
2	0.258	0.256	−0.78%	0.248	−3.88%	Translational vibration
3	0.299	0.306	2.34%	0.297	−0.67%	Torsional vibration
4	0.735	0.78	6.12%	0.757	2.99%	Bending vibration
5	0.807	0.8	−0.87%	0.773	−4.21%	Bending vibration
6	0.936	0.955	2.03%	0.925	−1.17%	Torsional vibration

## Data Availability

The original contributions presented in the study are included in the article, further inquiries can be directed to the corresponding author.

## Abbreviations

The following abbreviations are used in this manuscript:

CFST	Concrete-filled steel tubular
RAC-FST	Recycled aggregate concrete-filled steel tubular
CFRP	Carbon fiber-reinforced polymer
FRP	Fiber-reinforced polymer
ϕ	Stirrup diameter
*b*_f_	Width of steel beam flange
*f*_c_	Axial compressive strength of concrete
*f*_y_	Yield strength of the steel tube
*f*_cu_	Concrete cube strength
*η_r_*	Replacement rate
*ε*	Strain
PGA	Peak ground acceleration
